# Validation and Application of a Derivatization-Free RP-HPLC-DAD Method for the Determination of Low Molecular Weight Salivary Metabolites

**DOI:** 10.3390/ijerph17176158

**Published:** 2020-08-25

**Authors:** Beatrice Campanella, Tommaso Lomonaco, Edoardo Benedetti, Massimo Onor, Riccardo Nieri, Emilia Bramanti

**Affiliations:** 1 National Research Council of Italy, C.N.R., Institute of Chemistry of Organometallic Compounds-ICCOM, 56124 Pisa, Italy; beatrice.campanella@pi.iccom.cnr.it (B.C.); onor@pi.iccom.cnr.it (M.O.); riky.46@hotmail.it (R.N.); 2 Department of Chemistry and Industrial Chemistry, University of Pisa, 56124 Pisa, Italy; tommaso.lomonaco@unipi.it; 3 Hematology Unit, Department of Oncology, University of Pisa, 56100 Pisa, Italy; edobenedetti@gmail.com

**Keywords:** saliva, carboxylic acids, amino carboxylic acid, hydroxy organic acids, organic acids, HPLC-DAD multianalyte analysis

## Abstract

Saliva is an interesting, non-conventional, valuable diagnostic fluid. It can be collected using standardized sampling device; thus, its sampling is easy and non-invasive, it contains a variety of organic metabolites that reflect blood composition. The aim of this study was to validate a user-friendly method for the simultaneous determination of low molecular weight metabolites in saliva. We have optimized and validated a high throughput, direct, low-cost reversed phase liquid chromatographic method with diode array detection method without any pre- or post-column derivatization. We indexed salivary biomolecules in 35 whole non-stimulated saliva samples collected in 8 individuals in different days, including organic acids and amino acids and other carbonyl compounds. Among these, 16 whole saliva samples were collected by a single individual over three weeks before, during and after treatment with antibiotic in order to investigate the dynamics of metabolites. The concentrations of the metabolites were compared with the literature data. The multianalyte method here proposed requires a minimal sample handling and it is cost-effectiveness as it makes possible to analyze a high number of samples with basic instrumentation. The identification and quantitation of salivary metabolites may allow the definition of potential biomarkers for non-invasive “personal monitoring” during drug treatments, work out, or life habits over time.

## 1. Introduction

Low molecular weight metabolites are the final products of cellular processes, and their concentration in cells or biological fluids may reflect the response of biological systems to internal (e.g., enzyme activity and gene expression) or environmental factors (toxic agents) [1]. Most of these metabolites are characterized by the presence of the carboxylic functional group, such as short chain fatty acids (SCFAs), molecules of the tricarboxylic acid (TCA) cycle, dicarbonyl and hydroxycarbonyl compounds, ketone bodies and amino acids [2,3].

Branched-chain SCFAs are derived from the catabolism of branched-chain amino acids [4]. SCFAs and ketone bodies are relevant metabolites to assess human health status, since they have a recognized role in diseases as systemic inflammation, inflammatory bowel disease, obesity, diabetes and others [5,6,7,8,9]. SCFAs are also the primary end products of fermentation of non-digestible carbohydrates and they have been correlated with the metabolic syndrome and energy metabolism [10,11,12].

Ketone bodies (i.e., β-hydroxybutyrate, acetone, and acetoacetate) are mainly produced in the liver mitochondrion from the breakdown of acetyl-CoA, and they increase in metabolic situations such as starvation, endurance, malnutrition, and metabolic disorders including diabetes mellitus and chronic liver diseases [13]. They are produced also in the colon [6]. Recently, Crawford et al. found that ketogenesis regulates hepatic TCA cycle, glucose and lipid metabolism has a role in the development of nonalcoholic fatty liver disease [14]. Moreover, increasing evidences show that ketone bodies not only have a crucial role as alternative metabolic fuel source, but they play a pivotal part in mammalian metabolic pathways such as β-oxidation, in TCA cycle, gluconeogenesis, de novo lipogenesis, and biosynthesis of sterols [13,15].

The determination of lactate and pyruvate in biological fluids is a topic of the utmost importance in sport medicine to monitor the performance level of athletes, and in clinics in all diseases involving tissue hypoxia [16]. In the presence of a large intake of carbohydrates, fermentative bacteria of the lower intestine may give an overproduction of lactic acid that can be accumulated, absorbed in the systemic circulation and metabolized to pyruvate in liver and kidney.

Altered concentration of TCA compounds has been also detected in serum of dementia patients because of impairment of glucose metabolism pathways [17,18].

Thus, the metabolism of all these compounds (SCFAs, ketone bodies and TCA cycle compounds), coexisting in many matrices with amino acids, nucleic acid metabolites, vitamins and cofactors and other important small molecular weight metabolites, seem to be strictly interconnected. A good analytical method to simultaneously quantify low molecular weight metabolites in biological fluids can undoubtedly aid to understand their metabolic effect and physiological signaling function in health and disease.

Metabolomics of biological fluids, tissue/cellular extracts and cell culture media, based on liquid and gas chromatographic (LC-MS, GC-MS) and nuclear magnetic resonance (NMR) techniques, combined with multivariate data analysis tools is a powerful approach to investigate alterations in metabolic pathways following various perturbing events (e.g., disease states, drugs and nutrition) [19]. Theodoridis et al. have evidenced benefits and drawbacks [20,21] of the “*holistic*” metabolite profiling, which has expanded over the past few decades and hence has evolved through various stages.

Alternatively to *holistic* approaches, derivatization reactions for both GC and LC analysis are in general addressed to specific classes of compounds [22,23,24]. In this approach the clear advantage of increasing the specificity and sensitivity of the method is balanced by the drawbacks of time-consuming sample preparation, mostly the handling of toxic derivatization agents and the loss of simultaneous determination of multiple classes of analytes.

Organic acids are generally separated in complex matrices using expensive ion exclusion or reversed-phase columns [25] and determined with various detectors (MS, UV, fluorescence) after a derivatization step [6,26,27,28,29,30,31,32], by employment of ion pairing agents [33,34,35] or by on-line complexation with Cu(II) [36]. The direct determination of organic acids by reversed-phase high performance liquid chromatography (RP-HPLC) has been previously proposed because of its simplicity, rapidity and stability. Several studies are related to the direct determination of SCFAs in fruits, fruit juice, wine and plant extracts [37,38,39,40,41,42,43], honey [44], estuarine and marine samples [45], pharmaceutical materials [46], in vitro fermentation broths [23], and faces [47]. Few studies report about the determination of alfa-ketoacids [28,48,49,50,51,52] using ion exchange chromatography or derivatization techniques: these methods are addressed to specific classes of compounds.

At the same time no studies have been reported on the direct, simultaneous determination of SCFAs and other carbonyl compounds in human saliva. Saliva is an interesting, non-conventional, valuable diagnostic fluid, it can be collected using standardized sampling devices, thus its sampling is easy and non-invasive. Saliva reflects the composition of several compounds in blood [53] and it contains a variety of organic metabolites (e.g., amino acids, amines, carboxylic acids, proteins, carbohydrates) and inorganic compounds ([13] and references therein). Thus, it can be an important diagnostic medium for proteomics [54] and metabolomics, in diagnostic medicine, toxicology and drug monitoring [20,46,55,56,57,58,59,60,61,62]. Interesting works have been published related to saliva metabolomics in personalized medicine [63], in physiological stress [64], or as in inflammation status due to obesity [65,66].

The simultaneous, direct determination of various classes of compounds in saliva by RP-HPLC and UV detection is attractive, although challenging because of the complexity of the matrix.

The aim of this work is to propose and validate a high throughput, direct, easy HPLC method using a RP column and a diode-array detection system (RP-HPLC-DAD) that allowed the determination of 20 metabolites in saliva. The concentrations of the metabolites were compared with the literature data. The investigation on a single case was to show the potentiality of a low cost multianalyte method, in a non-invasive “personal monitoring” in order to investigate the dynamics of salivary metabolites as useful biomarkers for the study of gut microbiota and health status [67].

## 2. Materials and Methods

### 2.1. Chemicals

Phosphoric and sulphuric acid for HPLC analysis were employed (V800287 VETEC ≥85% Sigma-Aldrich, Milan, Italy). Methanol and acetonitrile (ACN) for RP-HPLC were purchased from Carlo Erba (Rodano, Italy). Preparation/dilution of samples and solutions was performed gravimetrically using ultrapure MilliQ water (18.2 MΩ cm^−1^ at 25 °C, Millipore, Bedford, MA, USA).

Standard solutions for HPLC (TraceCERT^®^, 1000 mg/L in water) were purchased from Sigma-Aldrich, Milan, Italy (see Table S1 Supporting Information). All compounds had purity higher than 98% and thus were used without any further purification. Analyte stock solutions were prepared by dissolving a weighed amount of the pure compound in deionized water or as indicated in Table S1 (Supplementary Materials) and stored at 4 °C up to 1 month. Working solutions were prepared daily by diluting their stock solution with MilliQ water. As an analyte-free “blank matrix” is not available the external calibrations (Table S2) have been performed in the eluent phase, which could be considered a sample-like matrix. Saliva is, indeed, diluted 1:5 in the same phase before the analysis.

All liquid solutions and saliva samples were stored in sterile polypropylene containers purchased from Eppendorf (Milan, Italy).

### 2.2. Study Subjects

Salivary biomolecules were indexed in 35 whole non-stimulated saliva samples collected in 8 individuals in different days, including organic acids and amino acids and other carbonyl compounds. Among these, 16 saliva samples were collected from a nominally healthy volunteer over three weeks, before, during and after the treatment with rifaximin, an antibiotic with anti-inflammatory effects and eubiotic properties in gut microbiota [1,68,69,70] and previously analysed by headspace GC-MS.

Eight nominally non-smoking healthy volunteers, colleagues at CNR were invited to participate to the study and were enrolled in this study. The study has been performed in accordance to the Declaration of Helsinki. Written informed consent was obtained from all volunteers who agreed to provide saliva samples. For each participant we obtained—via a questionnaire administered face-to-face—demographic data, gender and physiological, clinical, and lifestyle characteristics. Before the study, participants were trained for saliva collection. 

The participant population is described in Table 1 and it consisted of 2 men (RN and FZ code) and 5 women (E, LP, BC, CM, EB code) ranging in age from 26 to 60 yrs (mean age ± standard deviation, 46.3 ± 9.8 yrs) for a total of 19 saliva samples. From 4 participants, three saliva samples were collected daily over three consecutive days to investigate the intra-subject biological variability.

An additional 16 non-stimulated saliva samples from a nominally healthy volunteer (50 yrs female, marked as Rifaximin study) were analysed for the pilot application of the method for the assessment of the gut microbiota status. Rifaximin is indeed an eubiotic commonly employed to treat severe and light dysbiosis and to improve the gut status [1]. The samples were collected daily before (for three days), during the treatment with rifaximin (400 mg/day in the first and fifth day, 800 mg/day in the second, third and fourth day), and every 1–2 days during 14 days after the treatment [67].

### 2.3. Saliva Collection and Processing

For all participants saliva samples were collected at the same time of day (6:00 a.m. to 7:00 a.m.) to avoid fluctuation in the results due to the circadian saliva cycle, after at least 8 h of fasting or tooth brushing. Salivette^®^ swabs were kept in the mouth for 5 min, without chewing and after collection were immediately stored at −20 °C and kept frozen at −20 °C until the day of analysis.

Salivette^®^ (Sarstaedt, Germany) roll-shaped polyester swabs were used for saliva collection. Oral fluid can be sampled using several procedures [71]. In the case of non-stimulated samples, oral fluid may be collected by draining, spitting, suction and/or adsorption into swab. Salivary secretion can be stimulated by applying few drops of citric acid (0.1–0.2 M) directly onto the tongue, or letting the patient chew paraffin wax, parafilm, rubber bands or chewing gum. We previously demonstrated that saliva sampling may affect the determination of several metabolite [62]. For this reason, a specific sampling method must be established and uniformly applied in the study.

Prior to analysis, swabs were thawed at room temperature and then centrifuged at 4500× *g* for 10 min at 4 °C (Eppendorf™ 5804R Centrifuge).

Saliva contains about 0.1–1.5 mg/mL proteins [72], which could interfere with the analysis of low molecular weight metabolites and shorten column lifetime. Thus, before HPLC analysis saliva samples were diluted 5 or 10 times in 5 mM sulphuric acid, filtered using a 0.20 μm RC Mini-Uniprep (Agilent Technologies, Milan, Italy) filter and then injected in the HPLC system (V_inj_ = 5 μL).

### 2.4. Method Validation and Statistical Analysis

The analytical method validation was performed in accordance with the International Conference on Harmonisation (ICH) guideline Q2B and included an evaluation of limits of detection (LOD) and quantification (LOQ), calibration curves, recovery, intra-day and inter-day precision [73]. The linearity of the detection response for each compound was examined, and calibration curves were determined at 3–4 concentration levels of metabolites diluted in the eluent phase by plotting concentration against peak area and by applying the least squares method (Table S2). LOD and LOQ were calculated as 3.3 and 10 times, respectively, the standard deviation of blank signal divided by slope of the regression equation. Table S2 reports the fitting parameters, the correlation coefficients of the calibration plots and the LOD of the metabolites analyzed. All calibration curves are linear in the concentration range explored, which has been selected specifically for the determination of these metabolites in saliva based on data from the literature [17,51,52,55,74,75,76,77,78,79,80].

Recovery was estimated by spiking a saliva pool sample at 3–4 concentration levels (2–6 mM malic, 0.2–0.4 mM uric, 0.5–1 mM propionic, 2–4 mM butyric, 1–2 mM isobutyric, 0.25–0.5 mM succinic acid) or at one concentration level (0.324 mM pyruvic acid, 1 mM VAL, 0.336 mM lactic acid, 1.224 mM beta hydroxy butyric acid, 1.014 mM acetic acid, 0.81 mM propionic acid, 0.104 mM citric acid, 0.05 mM uric acid, 0.0976 mM GSH, 0.056 mM GSSG, 0.106 mM creatinine, 0.104 mM PHE, 0.0666 mM TRP, 0.0695 mM TYR, 0.315 mM malic acid, 0.69 mM acetoacetic acid, 1.94 mM formic acid, 0.035 mM fumaric acid, 1.182 mM succinic acid) processing the sample as described in the experimental part. Recovery was determined by comparing the analyte response in a biological sample that is spiked with the analyte and processed, with the response in a blank sample spiked with the same amount of analyte. The analysis of both solutions allowed us to rule out the matrix effect, as the difference between the calibration slopes were not significantly different.

Intra- and inter-day precision was expressed as coefficient of variation (CV%) of measurements performed on the unspiked samples in a single day and on three consecutive days, respectively. The analysis of saliva sample 4, 5 and 6 allowed us to estimate the inter-day biological variability and to compare it with the inter-day reproducibility of the analysis.

Data were entered in Excel (Microsoft Corp, Washington, USA) software for correlation analysis and one-way ANOVA analysis with the critical level for significance set at *p* < 0.05.

### 2.5. Analysis of Metabolites by RP-HPLC with UV Detection

An Agilent 1260 Infinity HPLC system (G1311B quaternary pump) equipped with a 1260 Infinity High Performance Degasser, a TCC G1316A thermostat, 1260ALS autosampler (G1329B) and UV/vis diode array (1260 DAD G4212B) was employed. The identification of metabolites was based on the comparison of the retention time and UV spectra of standard compounds. The 220 nm detection was selected to control the interference of high absorbing compounds. The chromatographic separation was carried out by Zorbax Phenyl-Hexyl RP C18 (Agilent Technology) 250 × 4.6 mm (silica particle size 4 μm) at 45 °C using the following elution profile: 15 min isocratic elution with 100% 5 mM sulphuric acid (pH 2.2), followed by 10 min gradient to 80% methanol and 10 min isocratic elution in 80% methanol (flow 0.8 mL/min). The column was rinsed with 100% methanol for 15 min and the re-equilibration step was performed. The same gradient was applied where indicated using 0.1% phosphoric acid in water. Detection was performed at 220 nm. All the solutions were filtered using a 0.22 μm regenerate cellulose filter (Millipore, Milan, Italy).

## 3. Results and Discussion

### 3.1. Method Validation in Saliva

**Figure 1:**
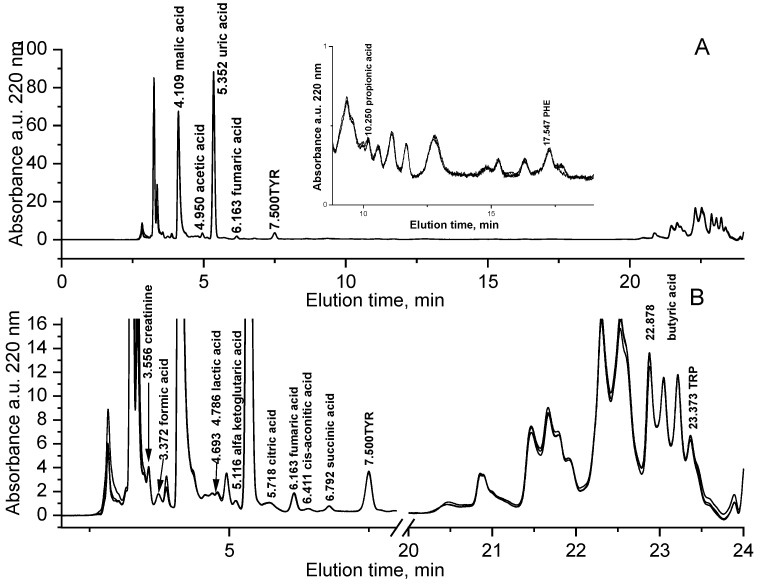
(**A**) Absorbance chromatograms at 220 nm of N = 3 replicates of a saliva sample n. 2 diluted (1:5) in 5 mM sulphuric acid (V_inj_ = 5 μL). Elution in 5 mM sulphuric acid (inlet: 8.8–19 min elution interval); (**B**) zoom of (**A**).

Figure 1 shows the superimposition of the absorbance chromatograms at 220 nm of three replicates of a saliva sample (from subject n.2) diluted 1:5 and a blank solution (5 mM sulphuric acid) analyzed in different days. The method herein proposed does not require any derivatization procedure and the samples have been straightforwardly diluted, filtered and analyzed.

In the chromatograms reported in Figure 1, 18 metabolites have been identified in saliva sample based on their retention time and UV spectrum. In order to confirm the assignment of the chromatographic peaks and to evaluate the accuracy of the method in the absence of certified reference materials, we analyzed a pooled saliva sample fortified with these 19 metabolites selected. Recovery was also evaluated for VAL and pyruvic acid, potentially present in saliva.

Table 2 shows the results obtained by recovery experiments. The mean recovery was 104% ranging between 70 (GSH) and 134% (GSSG). The low recovery of GSH and the corresponding over recovery of GSSG may be due to the oxidation of GSH itself spiked in the same sample.

Lactic acid elutes in saliva samples in two peaks at t_R_ = 4.648 min and 4.802 min. This feature has been previously observed [16,62] and it is currently under investigation. We hypothesize that the second peak of the lactic acid is probably due to the presence of a dimeric species of lactic acid. Lactate determination was validated by analyzing a sample set using the HPLC-UV method proposed and the derivatization method of lactate with 9-chloromethyl anthracene [16] (slope = 0.9771, R^2^ = 0.9761, Figure S1A). The results have been compared with the Bland–Altman plot (Figure S1B), a graphical method that compares the mean of the results of two techniques against the value of the difference. The limits of agreement were determined by taking 1.96 SD on either side of the bias. Since data were not normally distributed and not homoscedastic, a log10 transformation was performed before the comparison. As the figure shows, the scatter values are included in the acceptability range [81].

The good results of the recovery experiments guaranteed the suitability of external calibration. Despite the use of the external standardization for quantitation may be affected by instrument and sample conditions, the use of internal calibration for each sample would slow down the procedure, making the method poorly applicable to many samples. The low cost and the high throughput of this method is its strength, in view of the data treatment by chemometric techniques.

Analyte stability over the short and long term was evaluated with the analysis of two saliva samples. Each saliva sample was analyzed in triplicate over 8 h (day 1) and in triplicate after 48 h (day 2). Samples were kept at room temperature (21 ± 1 °C). In both cases, the variation coefficient respect to the first determination was within 15% for each analyte, as recommended by several guidelines for bioanalytical methods validation (i.e., US FDA and EMEA).

**Figure 2:**
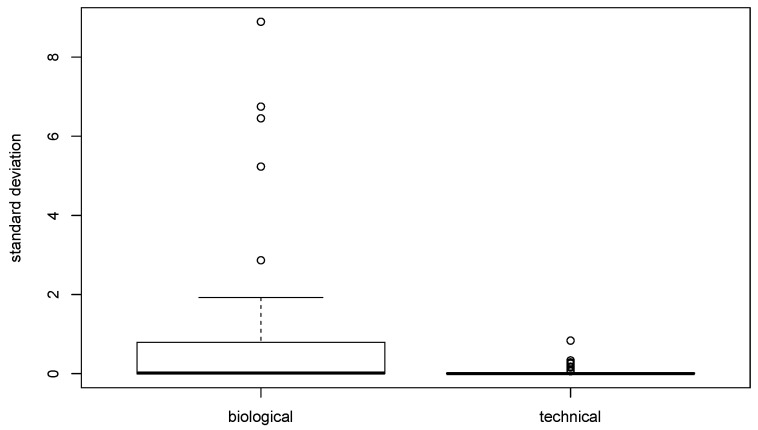
Standard deviation of all analytes for biological and technical variability, showing median standard deviation, interquartile ranges and outliers.

The biological variability was investigated in saliva from subjects n. 4, 5 and 6 sampled over 3 different consecutive days. A comparison was made between biological and technical variability by calculating the standard deviation for each analyte in subjects no. 4–5–6 analyzed over three days for biological variability, and in two subjects analyzed in triplicates (no. 2–4 pool) for technical variability. The results, compared as a box plot in Figure 2, show that the overall biological variability was markedly higher than the technical.

Tables S3–S6 report the complete statistics of the data.

### 3.2. Metabolites Quantification in Saliva Samples

Table 3 reports the statistics on the concentration of 20 metabolites identified and quantified in saliva samples collected from 7 subjects included in this study. Two main works have been published on the human saliva metabolome in the last years, based on NMR, GC–MS and LC–MS [55] and HPLC–UV analysis [75]. Other works are mentioned in Table 3 and in the Human Metabolome Data Base (HMDB) [82].

The values of Table 3 are mostly consistent with the normal concentration level of these metabolites in saliva of nominally healthy subjects (last column) [17,55,74,75,76,80,82]. However, several significant differences are herein discussed.

The complete data set is reported in the Supporting Information (Table S7).

Malic acid (t_R_ = 4.005 min) resulted as the main metabolite in saliva (24.0 ± 13.7 mM) (Figure S2A). Dihydrouracil (DHU) concentration has been reported to be 2168 ± 128 μM [55] and 2210 ± 353 μM by HPLC-UV analysis [75]. However, the analysis of standard solutions (Table S2) and the spike of DHU to saliva sample showed a peak eluting at 5.167 min excluding the presence of DHU in saliva (Figure S2B), in agreement with other authors that found DHU in saliva in the micromolar range or below [83,84,85]. 5-aminovaleric acid (or 5-aminopentanoic acid) is produced either endogenously or through bacterial catabolism of lysine by gut or oral microflora. Despite high levels of 5-aminovaleric acid in biofluids having been reported (470 ± 343 μM [55] and 119 ± 93 μM [75]), this work excluded its presence in saliva. The standard addition of 5-aminovaleric acid, eluting at 3.636 min, and of formic acid eluting at 3.723 min, confirmed the assignment of the peak at 3.736 min to formic acid (Figure S2C). Furthermore, 5-aminovaleric acid in [55,75] has been determined using NMR without a separation and it cannot be excluded that other saliva compounds may have analogous chemical-shifts.

Acetoacetic acid was < LOD (2.1 μM) in 10 out of 13 saliva samples, in agreement with literature data (10.7 ± 4.33 [55]). In 3 saliva samples it was more than 10 times higher. Higher values and highly variable values were observed also for fumaric acid and lactic acid. These differences between our data and those reported in the literature can be explained considering the variability associated with saliva sample. Age, sex, drugs, sampling time, or devices employed for sampling and external variables (e.g., smoke, drinks etc.) may modify the chemical composition of saliva samples, making it difficult to identify reference values, as reported for other biological specimens (e.g., blood and urine). To overcome these issues, saliva analysis can be proposed to perform longitudinal study (i.e., sampling overtime from the same subject) instead of cross-sectional study (i.e., data from a population at one specific point in time), reducing the inter-subject variability and then improving the reliability of saliva analysis.

In our elution conditions, pyruvic acid (t_R_ = 4.272) cannot be detected in saliva, despite its acceptable LOQ value, due to the masking of malic acid, which is present at mM concentration levels. A significant interference of pyruvic acid on the quantitation of malic acid can be excluded by its micromolar concentration levels reported in saliva (85 ± 95 μM [55]; 69 ±69 μM [75]; 37–93 μM [76]).

To the author’s best knowledge, this is the first study validating HPLC-DAD for the analysis of a wide range of low molecular weight salivary metabolites as alternative to time-consuming, labor-intensive analytical methods. All HPLC-UV methods based on the derivatization of specific classes of analytes make these methods not applicable for the simultaneous determination of different classes. LC-MS and LC-MS-MS are powerful tools commonly employed for the determination of small metabolites such as SCFAs and amino acids with high specificity with respect to classical optical detection method. The direct determination of SCFAs by ion exclusion and reversed phase LC-MS is possible only with post-column neutralization [86], and it requires a complex instrument setup not suitable for routine analysis, especially in clinical setting. On the other hand, matrix effects make LC-ESI-MS quantitation without the use of an isotopically-labeled internal standard often disputable [87]. As far as LC-MS/MS concerns, the availability of isotope-labelled standard, the high cost of equipment and reagents, the complexity of data processing, as well as the need of ion suppression make the analysis of small metabolites difficult.

### 3.3. Metabolite Monitoring during Rifaximin Antibiotic Treatment

We recently demonstrated that salivary metabolites might be a reliable “mirror” of gut metabolites, by investigating the dynamics of salivary volatile organic compounds (VOCs) in a subject over time during antibiotic intake [70]. To test the potentiality and sensitivity of RP-HPLC-UV method for microbiota-related investigations, we report here the application to the simultaneous determination of the major metabolites present in 16 saliva samples collected from a single healthy subject before, during and following antibiotic treatment.

**Figure 3:**
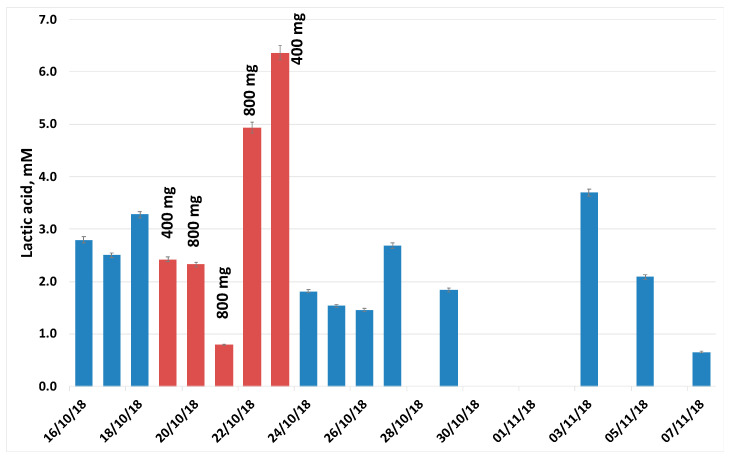
Representative trend of lactic acid (mM) during the experiment. The rifaximin dose (mg) taken the day before the sampling is reported (red bars).

Figure 3 shows the representative trend of lactic acid during the experiment. Table 4 reports the results of the quantitative analysis of 16 metabolites identified and quantified in the saliva samples from in this experiment. In these samples, α-ketoglutaric acid was also detected and quantified.

The concentration level of several metabolites (formic acid, α-ketoglutaric acid, uric, fumaric, succinic, propionic acid and PHE) show a significant decrease such as in the case of formic acid (during rifaximin vs. basal *p* = 0.051; after treatment vs. basal *p* = 0.012, multiple pairwise comparisons using Dunn’s procedure/two-tailed test:). For other metabolites concentration level (acetic, malic acid and TRP) we observe a decreasing trend after the treatment with rifaximin, although the statistical significance is not reached.

The trend of lactic acid determined by HLPC-DAD is analogous to the trend of ethanol previously reported and determined by headspace GC-MS [67]. The correlation analysis evidenced that, as we found in VOC analysis [67], the concentrations of many metabolites in saliva are significantly correlated (Figure S3). Table 5 reports representatively the correlation data (R^2^ and *p*-value, Pearson) of lactic acid with VAL, acetic acid, α-ketoglutaric acid, fumaric acid, succinic acid, TYR, PHE and TRP.

Lactic acid shows a strong positive correlation with acetic acid, the aromatic amino acids and α-ketoglutaric, succinic and fumaric acid, three key compounds of the tricarboxylic acid cycle. The meaning of these data, as well as their correlation with VOC results, is beyond the aim of this work, and involves the accurate analysis of complex metabolic pathways (in progress). However, these results encourage the integration of the results with GCMS data and to design more focused experiments that help their interpretation.

The reliability of the method here proposed has been further confirmed by the correlation analysis between analytes determinable by both HPLC-UV and headspace GC-MS, i.e., acetic and propionic acid. The correlation plot, shown in the Supporting Information (Figures S4 and S5, respectively), is characterized by R^2^ = 0.7657 and R^2^ = 0.8338 for acetic and propionic acid, respectively. No correlation was found for butyric acid, likely because of its lower volatility (boiling point = 163.7 °C for butyric, 141 °C for propionic and 117.9 °C for acetic acid) due to its lipophilicity (XLogP3 = 0.8 for butyric, 0.3 for propionic and -0.2 for acetic acid) [88].

These data confirm that saliva represents an interesting matrix reflecting gut microbiota *status*. The dynamic of low molecular weight salivary metabolites can be studied by a direct, fast, low-cost RP-HPLC-UV method.

Saliva analysis would indeed be advantageous with respect to the faecal analysis generally performed by culture-dependent methodologies, metagenomics [89], or LC and GC-MS techniques [21]. Although the sampling of faeces, and likewise saliva, is non-invasive, saliva sample analysis can be performed straightforwardly after a minimal sample handling. Finally, while faeces composition reflects only the last part of gut—in being the microbiota distributed differently in the various parts of gut—saliva reflects the “whole” gut status [90].

## 4. Conclusions

The application of saliva analysis for the metabolic profiling is appealing because of its easy sampling and storage. In this study, we propose a direct HPLC-DAD method for the simultaneous separation and quantification of 18- metabolites in saliva using an RP C18 column and UV detection at 220 nm. The method can be applied to the multianalyte determination of some of these metabolites in human saliva samples. This method does not require any derivatization procedure and it has been validated in human saliva after straightforward dilution and filtration (10–20 μL of saliva required as maximum amount of sampling volume). Recovery in saliva ranges between 86 and 121% (mean recovery 104%). 

The cost-effectiveness and minimal sample handling of the method here proposed make it possible to analyze a high number of samples and to employ “light” data processing to identify the key salivary metabolites as biomarkers for non-invasive continuous “personal monitoring” during drug treatments, work out, specific diets, or in disease states.

## Figures and Tables

**Table 1:** Description of the saliva samples and of the participant population.

Saliva Sample	Sex	yrs	Code	Annotations
1	F	51	E_030419	Single saliva sample
2	F	51	E_230319	Single saliva sample
3	F	51	E_310119	Single saliva sample
4	M	28	RN	3 saliva samples collected in 3 days consecutively
4pool	M	28	RN	Pool of 3 saliva samples collected in 3 days consecutively
5	M	48	FZ	3 saliva samples collected in 3 days consecutively
6	F	34	LP	3 saliva samples collected in 3 days consecutively
7	F	26	BC	Single saliva sample
8	F	60	CM	Single saliva sample
9	F	51	EB_030319	Single saliva sample
10	F	50	EB_060119	Single saliva sample
11	F	51	EB_090719	Single saliva sample
12	F	51	EB_290819	Single saliva sample
13	F	51	EB_030519	Single saliva sample
R1-R16	F	50	Rifaximin study	16 saliva sample collected in 16 days consecutively

yrs = years; F female; M male.

**Table 2:** Recovery experiments of metabolites in saliva sample n. 2 (N = 3 replicates).

Metabolite	T_R_ (min)	Slope (mM^−1^)	Intercept	R^2^	Conc_found_ in Saliva (mM)	Recovery (%)
Creatinine	3.603	8633	7.9	0.9999	0.005 ± 0.002	101.7
Formic acid	3.736	63	2	0.9974	0.169 ± 0.020	99.8
Malic acid	4.015	173	528.7	0.9999	16.4 ± 0.020	106.3
Pyruvic acid	4.240	1164	0	0.9999	<LOD	121.3
VAL	4.362	64	0	0.9899	<LOD	97.0
Lactic acid	4.648–4.802	111	3.8	0.9985	0.190 ± 0.020	100.6
Acetic acid	4.954	39	15.4	0.9673	2.270 ± 0.100	85.5
Uric acid	5.341	13,320	810	0.987	0.322 ± 0.020	104.3
Citric acid	5.510	412	0	0.9999	<LOD	119.3
GSH	5.950	1445	0	0.9999	<LOD	69.7
Fumaric acid	6.170	23,793	5.25	0.9999	0.0011 ± 0.001	113.6
Succinic acid	6.756	96	7.82	0.9981	0.4247 ± 0.030	108.8
Acetoacetic acid	7.040	70	0	0.9999	< LOD	99.4
Tyrosine	7.504	18,408	40.7	0.9996	0.0115 ± 0.010	110.1
GSSG	8.167	7214	0	0.9999	<LOD	133.5
Propionic acid	10.25	49	1.7	0.9975	0.1979 ± 0.020	95.2
Phenylalanine	17.547	3642	6.24	0.9999	0.0089 ± 0.001	109.4
Butyric acid	22.838	67 ± 7	4.9 ± 2.7	0.981	0.370 ± 0.040	101
Tryptophane	23.392	70,892	34.6	0.9999	0.0025 ± 0.002	110.8

**Table 3:** Statistics on μM concentration values of metabolites quantified in saliva samples form nominally healthy volunteers (saliva samples 1–13).

	N. Missing Values	Min	Max	Mean	SD (n−1)	Median	IQR	Literature Value (μM)
*Creatinine*	0	2.3	70.9	22.7	22.6	10.0	23.4	2- > 10 [74] 5 ± 3 [55] 6.5 ± 2 [17,75] 18–37 [76]
*Formic acid*	0	137.3	4687.8	1466.4	1442.8	1066.1	1420.6	7–244 [77] 600 ± 750 [51]
*Malic acid*	0	7290.1	57,645.1	24,038.7	13,731.4	23,367.3	19,916.7	6 ± 3 [55] 20 ± 11 [75] 20.45 ± 10.87 [17]
*VAL*	2	9.8	8303.0	2327.9	2452.0	2118.7	3326.6	16.2 ± 12.3 [78] 48 ± 34 [55] 4 ± 2 [75]
*Lactic acid*	0	190.0	8040.9	2467.7	2470.2	1637.9	2462.1	527 ± 690 [55] 511 ± 612 [75] 73–208 [76]
*Acetic acid*	0	14.1	22,898.6	4265.0	5968.6	2163.0	2279.3	6815 ± 4311 [55] 1000–1500 [52] 1200–3261 [76]
*α-ketoglutaric*	9	4.1	65.9	23.5	24.8	11.9	19.1	5.27 ± 3.61 [75]
*uric acid*	0	95.6	358.9	209.9	82.5	216.5	122.0	184 ± 22 [79] 179 ± 84 [80]
*Citric acid*	11	139.4	289.9	214.6	75.2	214.6	75.2	29 ± 10 [55] 18 (1–338) [75]
*GSH*	10	32.8	44.1	37.0	5.1	34.0	5.7	7 ± 6 [55]
*Fumaric acid*	0	1.1	85.7	18.2	26.8	7.4	10.2	2 ± 0.7 [55] 1 ± 0.5 [75]
*cis-aconitic acid*	9	0.7	25.9	7.4	10.7	1.5	6.5	3 ± 1 [17,75]
*Succinic acid*	3	51.1	4755.7	946.9	1341.0	345.9	928.5	125 ± 181 [55] 2260 (60–4460) [75] 8–21 [76]
*Acetoacetic acid*	10	164.3	823.8	593.7	303.9	792.9	329.8	10.7 ± 4.33 [55]
*TYR*	0	3.6	81.0	26.1	23.9	11.5	32.9	40 ± 25 [55] 36 ± 15 [75]
*GSSG*	5	6.6	56.2	18.4	16.3	9.4	14.1	0.9 ± 0.4 [17]
*Propionic acid*	0	12.7	6990.2	1018.5	1797.4	333.3	1078.5	1412 ± 1090 [55] 1180 ± 1270 [75] 6.5–338 [76]
*PHE*	0	0.2	233.0	41.8	59.9	14.3	32.6	44 ± 23 [55] 18 (1–112) [75]
*Butyric acid*	0	9.8	13,621.2	2916.8	3635.7	2189.4	4171.7	277 ± 199 [55] 1470 (0–2940) [75]
*TRP*	0	0.5	16.2	6.2	5.5	2.7	9.1	4.8 ± 3 [55] 0.5 ± 0.5 [75]

**Table 4:** Minimum and maximum value, and mean concentration (μM) of the selected metabolites in 16 saliva samples collected from a single healthy subject before (N = 3), during (N = 5) and following (N = 8) antibiotic treatment.

	Min	Max	Mean	SD (n−1)	CV% (n−1)	Median	IQR
Creatinine (basal)	2.9	7.1	4.7	2.1	46.2	3.9	2.1
During rifaximin	3.7	6.6	4.7	1.1	23.3	4.6	0.6
After rifaximin	4.0	5.9	4.8	0.7	14.2	4.7	1.1
Formic acid (basal)	206.3	317.5	254.0	57.2	22.5	238.1	55.6
During rifaximin	39.7	214.3	131.7	73.9	56.1	150.8	111.1
After rifaximin	35.7	198.4	112.5	62.7	55.7	107.1	85.5
Malic acid (basal)	24736.1	26234.6	25426.5	756.1	3.0	25308.6	749.2
During rifaximin	13765.4	42098.8	23364.2	10861.2	46.5	20648.1	1234.6
After rifaximin	13688.3	29876.5	18776.2	5416.4	28.8	16354.9	6159.7
VAL (basal)	833.3	1136.4	1,007.6	156.5	15.5	1053.0	151.5
During rifaximin	818.2	5,401.5	2,195.5	1941.1	88.4	1166.7	1742.4
After rifaximin	818.2	3,712.1	1,747.2	993.2	56.8	1284.1	1075.8
Lactic acid (basal)	2500.0	3272.7	2856.1	389.9	13.7	2795.5	386.4
During rifaximin	790.9	6363.6	3367.3	2239.5	66.5	2422.7	2613.6
After rifaximin	650.0	3695.5	1969.3	905.2	46.0	1825.0	719.3
Acetic acid (basal)	1847.8	2717.4	2235.5	442.4	19.8	2141.3	434.8
During rifaximin	847.8	3815.2	1997.8	1103.1	55.2	1728.3	423.9
After rifaximin	1130.4	2260.9	1773.1	380.7	21.5	1722.8	418.5
α-ketoglutaric acid (basal)	103.6	144.3	125.5	20.5	16.4	128.4	20.4
During rifaximin	39.6	215.4	113.0	64.7	57.3	105.1	34.8
After rifaximin	12.2	103.6	66.4	27.0	40.7	63.8	18.4
Uric acid (basal)	289.7	298.3	294.5	4.4	1.5	295.5	4.3
During rifaximin	13.7	295.5	181.4	136.5	75.2	263.8	228.8
After rifaximin	12.0	334.9	166.5	117.7	70.6	194.2	183.2
GSH (basal)	12.3	14.5	13.7	1.2	8.7	14.2	1.1
During rifaximin	13.0	19.5	17.3	2.6	15.0	18.1	2.2
After rifaximin	0.7	21.2	11.8	6.8	58.1	13.1	5.4
Fumaric acid (basal)	17.9	20.3	19.0	1.3	6.6	18.6	1.2
During rifaximin	5.8	29.8	16.2	8.7	53.4	15.5	3.1
After rifaximin	3.1	14.5	9.5	3.3	35.1	9.8	2.3
Succinic acid (basal)	1022.7	1096.6	1056.8	37.3	3.5	1051.1	36.9
During rifaximin	221.6	1340.9	750.0	455.3	60.7	642.0	613.6
After rifaximin	187.5	858.0	573.2	227.5	39.7	579.5	268.5
TYR (basal)	6.7	8.9	7.7	1.1	14.6	7.5	1.1
During rifaximin	6.0	19.3	10.9	5.2	48.2	9.3	4.5
After rifaximin	5.2	11.7	8.6	2.6	30.4	8.2	4.5
Propionic acid (basal)	245.1	294.1	264.7	25.9	9.8	254.9	24.5
During rifaximin	34.3	281.4	148.2	109.3	73.7	156.9	182.4
After rifaximin	49.0	134.3	93.3	33.9	36.3	86.3	60.8
PHE (basal)	19.2	22.2	20.6	1.5	7.4	20.3	1.5
During rifaximin	8.1	39.7	21.0	12.2	57.8	17.9	11.3
After rifaximin	8.6	19.2	13.6	4.1	30.0	13.4	4.8
Butyric acid (basal)	1363.6	1515.2	1441.9	75.9	5.3	1447.0	75.8
During rifaximin	1204.5	2030.3	1527.3	378.4	24.8	1325.8	590.9
After rifaximin	424.2	1924.2	1218.8	550.8	45.2	1197.0	611.7
TRP (basal)	2.0	2.5	2.2	0.3	12.3	2.3	0.3
During rifaximin	1.3	4.2	2.4	1.2	49.3	1.8	1.4
After rifaximin	0.4	2.4	1.5	0.6	41.0	1.5	0.5

**Table 5:** Coefficients of determination (R^2^) and *p*-values (Pearson) for the correlation of lactic acid with other metabolites found in in saliva samples from the experiment with rifaximin.

	R^2^	*p*-Value (Pearson)
VAL	0.6339	0.0002
Acetic acid	0.5915	0.0005
a-ketoglutaric acid	0.6436	0.0002
Fumaric acid	0.6601	0.0001
Succinic acid	0.6021	0.0004
TYR	0.4554	0.0041
PHE	0.6099	0.0004
TRP	0.4196	0.0067

## Supplementary Materials

The following are available online at https://www.mdpi.com/1660-4601/17/17/6158/s1, Table S1. Sigma-Aldrich product codes of standard products employed; Table S2. Retention times, fitting parameters (slope and standard deviation SD of the slope), correlation coefficients of the calibration plots and limit of detection (LOD) of the selected metabolites analyzed (V_inj_ = 5 μL; N = 3 replicates). Table S3. Statistics on the compounds identified and quantified (mM) in saliva sample of subject n. 2 (intra-day reproducibility test, N = 3) of Figure 1; Table S4. Statistics on the compounds identified and quantified (mM) in saliva pool sample (inter-day reproducibility test, N = 3) (subject n. 4 pool); Table S5. Statistics on the compounds identified and quantified (mM) in saliva samples (N = 3) (subject n. 4); Table S6. Statistics on the compounds identified and quantified (mM) in saliva samples (N = 3) (subject n. 5); Table S7. μM concentration values of metabolites quantified in 13 different saliva samples form nominally healthy volunteers; Figure S1. Determination of lactic acid in saliva by 9-CMA method vs. HPLC-UV method; Figure S2. (A) Absorbance chromatogram at 220 nm of unspiked saliva (saliva pool n. 4) and spiked with 2, 4 and 6 mM malic acid. (B) Absorbance chromatogram at 220 nm of unspiked saliva (saliva pool n. 4) and spiked with 1 mM dihydrouracil. (C) Absorbance chromatogram at 220 nm of unspiked saliva (saliva pool n. 4) and spiked and 0.5 mM 5-amino valeric acid (dotted line) or 0.5 mM formic acid (dash-dot line); Figure S3 Correlation plot for 16 metabolites quantified in saliva samples from the experiment with rifaximin (correlation obtained after data scaling); Figure S4. Determination of acetic acid in saliva by HS-GCMS method vs. HPLC-UV method; Figure S5. Determination of propionic acid in saliva by HS-GCMS method vs. HPLC-UV method.

## References

[1] Ponziani F.R., Scaldaferri F., Petito V., Sterbini F.P., Pecere S., Lopetuso L.R., Palladini A., Gerardi V., Masucci L., Pompili M. (2016). The Role of Antibiotics in Gut Microbiota Modulation: The Eubiotic Effects of Rifaximin. Dig. Dis..

[2] Olson K.A., Schell J.C., Rutter J. (2016). Pyruvate and Metabolic Flexibility: Illuminating a Path Toward Selective Cancer Therapies. Trends Biochem. Sci..

[3] Gibala M.J., Young M.E., Taegtmeyer H. (2000). Anaplerosis of the citric acid cycle: Role in energy metabolism of heart and skeletal muscle. Acta Physiol. Scand..

[4] Zheng X., Qiu Y., Zhong W., Baxter S., Su M., Li Q., Xie G., Ore B.M., Qiao S., Spencer M.D. (2013). A targeted metabolomic protocol for short-chain fatty acids and branched-chain amino acids. Metabolomics.

[5] Tan J., McKenzie C., Potamitis M., Thorburn A.N., Mackay C.R., Macia L., Frederick W.A. (2014). Chapter Three—The Role of Short-Chain Fatty Acids in Health and Disease.

[6] Zeng M., Cao H. (2018). Fast quantification of short chain fatty acids and ketone bodies by liquid chromatography-tandem mass spectrometry after facile derivatization coupled with liquid-liquid extraction. J. Chromatogr. B.

[7] Ghimenti S., Tabucchi S., Lomonaco T., Di Francesco F., Fuoco R., Onor M., Lenzi S., Trivella M.G. (2013). Monitoring breath during oral glucose tolerance tests. J. Breath Res..

[8] Biagini D., Lomonaco T., Ghimenti S., Bellagambi F.G., Onor M., Scali M.C., Barletta V., Marzilli M., Salvo P., Trivella M.G. (2017). Determination of volatile organic compounds in exhaled breath of heart failure patients by needle trap micro-extraction coupled with gas chromatography-tandem mass spectrometry. J. Breath Res..

[9] Lomonaco T., Romani A., Ghimenti S., Biagini D., Bellagambi F.G., Onor M., Salvo P., Fuoco R., Di Francesco F. (2018). Determination of carbonyl compounds in exhaled breath by on-sorbent derivatization coupled with thermal desorption and gas chromatography-tandem mass spectrometry. J. Breath Res..

[10] Hu J., Lin S., Zheng B., Cheung P.C.K. (2018). Short-chain fatty acids in control of energy metabolism. Crit. Rev. Food Sci. Nutr..

[11] Canfora E.E., Jocken J.W., Blaak E.E. (2015). Short-chain fatty acids in control of body weight and insulin sensitivity. Nat. Rev. Endocrinol..

[12] Koh A., De Vadder F., Kovatcheva-Datchary P., Bäckhed F. (2016). From Dietary Fiber to Host Physiology: Short-Chain Fatty Acids as Key Bacterial Metabolites. Cell.

[13] Puchalska P., Crawford P.A. (2017). Multi-dimensional Roles of Ketone Bodies in Fuel Metabolism, Signaling, and Therapeutics. Cell Metab..

[14] Cotter D.G., Ercal B., Huang X., Leid J.M., D’Avignon D.A., Graham M.J., Dietzen D.J., Brunt E.M., Patti G.J., Crawford P.A. (2014). Ketogenesis prevents diet-induced fatty liver injury and hyperglycemia. J. Clin. Investig..

[15] Newman J.C., Verdin E. (2014). Ketone bodies as signaling metabolites. Trends Endocrinol. Metab..

[16] Pellegrini D., Onor M., Degano I., Bramanti E. (2014). Development and validation of a novel derivatization method for the determination of lactate in urine and saliva by liquid chromatography with UV and fluorescence detection. Talanta.

[17] Tsuruoka M., Hara J., Hirayama A., Sugimoto M., Soga T., Shankle W.R., Tomita M. (2013). Capillary electrophoresis-mass spectrometry-based metabolome analysis of serum and saliva from neurodegenerative dementia patients. Electrophoresis.

[18] Wilkins J.M., Trushina E. (2018). Application of Metabolomics in Alzheimer’s Disease. Front. Neurol..

[19] Brennan L. (2013). Metabolomics in nutrition research: Current status and perspectives. Biochem. Soc. Trans..

[20] Gika H.G., Zisi C., Theodoridis G., Wilson I.D. (2016). Protocol for quality control in metabolic profiling of biological fluids by U(H)PLC-MS. J. Chromatogr. B.

[21] Theodoridis G.A., Gika H.G., Want E.J., Wilson I.D. (2012). Liquid chromatography–mass spectrometry based global metabolite profiling: A review. Anal. Chim. Acta.

[22] Ferreira A.M.C., Laespada M.E.F., Pavón J.L.P., Cordero B.M. (2013). In situ aqueous derivatization as sample preparation technique for gas chromatographic determinations. J. Chromatogr. A.

[23] De Baere S., Eeckhaut V., Steppe M., De Maesschalck C., De Backer P., Van Immerseel F., Croubels S. (2013). Development of a HPLC–UV method for the quantitative determination of four short-chain fatty acids and lactic acid produced by intestinal bacteria during in vitro fermentation. J. Pharm. Biomed. Anal..

[24] Ewaschuk J.B., Zello G.A., Naylor J.M., Brocks D.R. (2002). Metabolic acidosis: Separation methods and biological relevance of organic acids and lactic acid enantiomers. J. Chromatogr. B.

[25] Kotani A., Miyaguchi Y., Kohama M., Ohtsuka T., Shiratori T., Kusu F. (2009). Determination of short-chain fatty acids in rat and human feces by high-performance liquid chromatography with electrochemical detection. Anal. Sci..

[26] Mochizuki Y., Inagaki S., Suzuki M., Min J.Z., Inoue K., Todoroki K., Toyo’Oka T. (2013). A novel derivatization reagent possessing a bromoquinolinium structure for biological carboxylic acids in HPLC-ESI-MS/MS. J. Sep. Sci..

[27] Marquis B.J., Louks H.P., Bose C., Wolfe R.R., Singh S.P. (2017). A New Derivatization Reagent for HPLC-MS Analysis of Biological Organic Acids. Chromatographia.

[28] Guo X.F., Li Y., Wang H., Zhang H.S. (2014). Determination of fatty acids in saliva of smokers and nonsmokers by HPLC with fluorescence detection using a hydrazine-based difluoro-boraindacene reagent. Chromatographia.

[29] Mukherjee P.S., Karnes H.T. (1996). Ultraviolet and fluorescence derivatization reagents for carboxylic acids suitable for high performance liquid chromatography: A review. Biomed. Chromatogr..

[30] Kubota K., Fukushima T., Yuji R., Miyano H., Hirayama K., Santa T., Imai K. (2005). Development of an HPLC-fluorescence determination method for carboxylic acids related to the tricarboxylic acid cycle as a metabolome tool. Biomed. Chromatogr..

[31] Johnson D.W. (2005). Contemporary clinical usage of LC/MS: Analysis of biologically important carboxylic acids. Clin. Biochem..

[32] Gikas E., Derventi M., Panderi I., Vavayannis A., Kazanis M., Parissi-Poulou M. (2002). A new fluorogenic reagent for labelling carboxylic acids in HPLC. J. Liq. Chromatogr. Relat. Technol..

[33] Kiefer P., Delmotte N., Vorholt J.A. (2011). Nanoscale Ion-Pair Reversed-Phase HPLC−MS for Sensitive Metabolome Analysis. Anal. Chem..

[34] Buescher J.M., Moco S., Sauer U., Zamboni N. (2010). Ultrahigh Performance Liquid Chromatography−Tandem Mass Spectrometry Method for Fast and Robust Quantification of Anionic and Aromatic Metabolites. Anal. Chem..

[35] Lu W., Clasquin M.F., Melamud E., Amador-Noguez D., Caudy A.A., Rabinowitz J.D. (2010). Metabolomic Analysis via Reversed-Phase Ion-Pairing Liquid Chromatography Coupled to a Stand Alone Orbitrap Mass Spectrometer. Anal. Chem..

[36] Kemmei T., Kodama S., Yamamoto A., Inoue Y., Hayakawa K. (2015). Reversed phase liquid chromatographic determination of organic acids using on-line complexation with copper(II) ion. Anal. Chim. Acta.

[37] Marcé R.M., Calull M., Manchobas R.M., Borrull F., Rius F.X. (1990). An optimized direct method for the determination of carboxylic acids in beverages by HPLC. Chromatographia.

[38] Tusseau D., Benoit C. (1987). Routine high-performance liquid chromatographic determination of carboxylic acids in wines and champagne. J. Chromatogr. A.

[39] Scherer R., Rybka A.C.P., Ballus C.A., Meinhart A.D., Filho J.T., Godoy H.T. (2012). Validation of a HPLC method for simultaneous determination of main organic acids in fruits and juices. Food Chem..

[40] de Quirós A.R.-B., Lage-Yusty M.A., López-Hernández J. (2009). HPLC analysis of organic acids using a novel stationary phase. Talanta.

[41] Pereira V., Câmara J.S., Cacho J., Marques J.C. (2010). HPLC-DAD methodology for the quantification of organic acids, furans and polyphenols by direct injection of wine samples. J. Sep. Sci..

[42] Shui G., Leong L.P. (2002). Separation and determination of organic acids and phenolic compounds in fruit juices and drinks by high-performance liquid chromatography. J. Chromatogr. A.

[43] Zong Y., Lin J., Xu H., Jia Z., Yang X., Choi M.M.F. (2015). Optimization and validation of an HPLC-photodiode array detector method for determination of organic acids in vinegar. J. AOAC Int..

[44] Suarez-Luque S., Mato I., Huidobro J.F., Simal-Lozano J., Sancho M.T. (2002). Rapid determination of minority organic acids in honey by high-performance liquid chromatography. J. Chromatogr. A.

[45] Edelkraut F., Brockmann U. (1990). Simulataneous determination of carboxylic acids and carbonyl compounds in estuaries by HPLC. Chromatographia.

[46] Liang Q., Liu H., Li X., Zhang A.H. (2016). High-throughput metabolomics analysis discovers salivary biomarkers for predicting mild cognitive impairment and Alzheimer′s disease. RSC Adv..

[47] Torii T., Kanemitsu K., Wada T., Itoh S., Kinugawa K., Hagiwara A. (2010). Measurement of short-chain fatty acids in human faeces using high-performance liquid chromatography: Specimen stability. Ann. Clin. Biochem..

[48] Tsuchiya H., Hashizume I., Tokunaga T., Tatsumi M., Takagi N., Hayashi T. (1983). High-performance liquid chromatography of α-keto acids in human saliva. Arch. Oral Biol..

[49] Tsutsui H., Mochizuki T., Maeda T., Noge I., Kitagawa Y., Min J.Z., Todoroki K., Inoue K., Toyo T. (2012). Simultaneous determination of DL-lactic acid and DL-3-hydroxybutyric acid enantiomers in saliva of diabetes mellitus patients by high-throughput LC-ESI-MS/MS. Anal. Bioanal. Chem..

[50] Stein J., Kulemeier J., Lembcke B., Caspary W.F. (1992). Simple and rapid method for determination of short-chain fatty acids in biological materials by high-performance liquid chromatography with ultraviolet detection. J. Chromatogr..

[51] Park Y.D., Jang J.H., Oh Y.J., Kwon H.J. (2014). Analyses of organic acids and inorganic anions and their relationship in human saliva before and after glucose intake. Arch. Oral Biol..

[52] Beighton D., Brailsford S.R., Gilbert S.C., Clark D.T., Rao S., Wilkins J.C., Tarelli E., Homer K.A. (2004). Intra-Oral Acid Production Associated with Eating Whole or Pulped Raw Fruits. Caries Res..

[53] Lima D.P., Diniz D.G., Moimaz S.A.S., Sumida D.H., Okamoto A.C. (2010). Saliva: Reflection of the body. Int. J. Infect. Dis..

[54] Khurshid Z., Zohaib S., Najeeb S., Zafar M.S., Slowey P.D., Almas K. (2016). Human Saliva Collection Devices for Proteomics: An Update. Int. J. Mol. Sci..

[55] Dame Z.T., Aziat F., Mandal R., Krishnamurthy R., Bouatra S., Borzouie S., Guo A.C., Sajed T., Deng L., Lin H. (2015). The human saliva metabolome. Metabolomics.

[56] Cuevas-Cordoba B., Santiago-Garcia J. (2014). Saliva: A Fluid of Study for OMICS. Omi. J. Integr. Biol..

[57] Michalke B., Rossbach B., Göen T., Schäferhenrich A., Scherer G., Hartwig A., MAK Commission (2016). Saliva as a matrix for human biomonitoring in occupational and environmental medicine [Biomonitoring Methods, 2015]. MAK Collect. Occup. Health Saf..

[58] Biagi S., Ghimenti S., Onor M., Bramanti E. (2012). Simultaneous determination of lactate and pyruvate in human sweat using reversed-phase high-performance liquid chromatography: A noninvasive approach. Biomed. Chromatogr..

[59] Bessonneau V., Boyaci E., Maciazek-Jurczyk M., Pawliszyn J. (2015). In vivo solid phase microextraction sampling of human saliva for non-invasive and on-site monitoring. Anal. Chim. Acta.

[60] Bonne N.J., Wong D.T.W. (2012). Salivary biomarker development using genomic, proteomic and metabolomic approaches. Genome Med..

[61] Lomonaco T., Ghimenti S., Piga I., Biagini D., Onor M., Fuoco R., Di Francesco F. (2014). Influence of Sampling on the Determination of Warfarin and Warfarin Alcohols in Oral Fluid. PLoS ONE.

[62] Lomonaco T., Ghimenti S., Biagini D., Bramanti E., Onor M., Bellagambi F.G., Fuoco R., Di Francesco F. (2018). The effect of sampling procedures on the urate and lactate concentration in oral fluid. Microchem. J..

[63] Beger R.D., Dunn W., Schmidt M.A., Gross S.S., Kirwan J.A., Cascante M., Brennan L., Wishart D.S., Oresic M., Hankemeier T. (2016). Metabolomics enables precision medicine: “A White Paper, Community Perspective”. Metabolomics.

[64] Malkar A., Devenport N.A., Martin H.J., Patel P., Turner M.A., Watson P., Maughan R.J., Reid H.J., Sharp B.L., Thomas C.L.P. (2013). Metabolic profiling of human saliva before and after induced physiological stress by ultra-high performance liquid chromatography-ion mobility-mass spectrometry. Metabolomics.

[65] Rangel-Huerta O.D., Pastor-Villaescusa B., Gil A. (2019). Are we close to defining a metabolomic signature of human obesity? A systematic review of metabolomics studies. Metabolomics.

[66] Zhang Z., Hong Y., Chen M., Tan N., Liu S., Nie X., Zhou W. (2020). Serum metabolomics reveals metabolic profiling for women with hyperandrogenism and insulin resistance in polycystic ovary syndrome. Metabolomics.

[67] Campanella B., Onor M., Lomonaco T., Benedetti E., Bramanti E. (2019). HS-SPME-GC-MS approach for the analysis of volatile salivary metabolites and application in a case study for the indirect assessment of gut microbiota. Anal. Bioanal. Chem..

[68] Ponziani F.R., Pompili M., Gasbarrini A. (2017). Rifaximin Re-treatment in Patients with Irritable Bowel Syndrome: Feels Like the First Time?. Dig. Dis. Sci..

[69] Ponziani F.R., Pecere S., Lopetuso L., Scaldaferri F., Cammarota G., Gasbarrini A. (2016). Rifaximin for the treatment of irritable bowel syndrome—A drug safety evaluation. Expert Opin. Drug Saf..

[70] Ponziani F.R., Gerardi V., Pecere S., D’Aversa F., Lopetuso L., Zocco M.A., Pompili M., Gasbarrini A. (2015). Effect of rifaximin on gut microbiota composition in advanced liver disease and its complications. World J. Gastroenterol..

[71] Navazesh M. (1993). Methods for Collecting Saliva. Ann. N. Y. Acad. Sci..

[72] Goodson J.M., Kantarci A., Hartman M.-L., Denis G.V., Stephens D., Hasturk H., Yaskell T., Vargas J., Wang X., Cugini M. (2014). Metabolic Disease Risk in Children by Salivary Biomarker Analysis. PLoS ONE.

[73] Variuos Authors (2005). Validation of Analytical Procedures: Text and Methodology Q2(R1). Proceedings of the International Conference on Harmonisation of Technical Requirements for Registration of Pharmaceuticals for Human Use.

[74] Silwood C.J.L., Lynch E., Claxson A.W.D., Grootveld M.C. (2002). 1H and (13)C NMR spectroscopic analysis of human saliva. J. Dent. Res..

[75] Sugimoto M., Saruta J., Matsuki C., To M., Onuma H., Kaneko M., Soga T., Tomita M., Tsukinoki K. (2013). Physiological and environmental parameters associated with mass spectrometry-based salivary metabolomic profiles. Metabolomics.

[76] Figueira J., Gouveia-Figueira S., Öhman C., Lif Holgerson P., Nording M.L., Öhman A. (2017). Metabolite quantification by NMR and LC-MS/MS reveals differences between unstimulated, stimulated, and pure parotid saliva. J. Pharm. Biomed. Anal..

[77] Takeda I., Stretch C., Barnaby P., Bhatnager K., Rankin K., Fu H., Weljie A., Jha N., Slupsky C. (2009). Understanding the human salivary metabolome. NMR Biomed..

[78] Nakamura Y., Kodama H., Satoh T., Adachi K., Watanabe S., Yokote Y., Sakagami H. (2010). Diurnal Changes in Salivary Amino Acid Concentrations. Vivo Brooklyn.

[79] Kochanska B., Smolenski R.T., Knap N. (2000). Determination of adenine nucleotides and their metabolites in human saliva. Acta Biochim. Pol..

[80] Riis J.L., Bryce C.I., Matin M.J., Stebbins J.L., Kornienko O., van Huisstede L., Granger D.A. (2018). The validity, stability, and utility of measuring uric acid in saliva. Biomark. Med..

[81] Choi S.W., Lam D.M.H. (2016). A little less conversation. Anaesthesia.

[82] Wishart D.S., Feunang Y.D., Marcu A., Guo A.C., Liang K., Vazquez-Fresno R., Sajed T., Johnson D., Li C., Karu N. (2018). HMDB 4.0: The human metabolome database for 2018. Nucleic Acids Res..

[83] Carlsson G., Odin E., Gustavsson B., Wettergren Y. (2014). Pretherapeutic uracil and dihydrouracil levels in saliva of colorectal cancer patients are associated with toxicity during adjuvant 5-fluorouracil-based chemotherapy. CANCER Chemother. Pharmacol..

[84] Andrade Galarza A.F., Linden R., Antunes M.V., Hahn R.Z., Raymundo S., da Silva A.C., Staggemeier R., Spilki F.R., Schwartsmann G. (2016). Endogenous plasma and salivary uracil to dihydrouracil ratios and DPYD genotyping as predictors of severe fluoropyrimidine toxicity in patients with gastrointestinal malignancies. Clin. Biochem..

[85] Antunes M.V., Raymundo S., Cezimbra da Silva A.C., Muller V.V., Vicente Neto O.J., Schwartsmann G., Linden R. (2019). Determination of Endogenous Concentrations of Uracil and Dihydrouracil in Dried Saliva Spots by LC-MS/MS. Ther. Drug Monit..

[86] van Eijk H.M.H., Bloemen J.G., Dejong C.H.C. (2009). Application of liquid chromatography–mass spectrometry to measure short chain fatty acids in blood. J. Chromatogr. B.

[87] Biagini D., Lomonaco T., Ghimenti S., Fusi J., Cerri E., De Angelis F., Bellagambi F.G., Oger C., Galano J.M., Bramanti E. (2020). Saliva as a non-invasive tool for monitoring oxidative stress in swimmers athletes performing a VO2max cycle ergometer test. Talanta.

[88] Kim S., Chen J., Cheng T., Gindulyte A., He J., He S., Li Q., Shoemaker B.A., Thiessen P.A., Yu B. (2019). PubChem 2019 update: Improved access to chemical data. Nucleic Acids Res..

[89] Panek M., Paljetak H.C., Baresic A., Peric M., Matijasic M., Lojkic I., Bender D.V., Krznaric Z., Verbanac D. (2018). Methodology challenges in studying human gut microbiota—Effects of collection, storage, DNA extraction and next generation sequencing technologies. Sci. Rep..

[90] Zmora N., Zilberman-Schapira G., Suez J., Mor U., Dori-Bachash M., Bashiardes S., Kotler E., Zur M., Regev-Lehavi D., Brik R.B.-Z. (2018). Personalized Gut Mucosal Colonization Resistance to Empiric Probiotics Is Associated with Unique Host and Microbiome Features. Cell.

