# Supplementary material for: Validation and Application of a Derivatization-Free RP-HPLC-DAD Method for the Determination of Low Molecular Weight Salivary Metabolites
Source: Int J Environ Res Public Health. 2020 Aug 25;17(17):6158. doi: 10.3390/ijerph17176158 (PMC7503734; doi:10.3390/ijerph17176158)
Supplement: Supplementary file 1 [file ijerph-17-06158-s001.pdf]

SUPPLEMENTARY MATERIAL to

Validation and Application of a Derivatization-Free RP-HPLC-DAD Method for the Determination of Low Molecular Weight Salivary Metabolites

Beatrice Campanella [1], Tommaso Lomonaco [2], Edoardo Benedetti [3], Massimo Onor [1], Riccardo Nieri [1] and Emilia Bramanti [1,*]

[1] National Research Council of Italy, C.N.R., Institute of Chemistry of Organometallic Compounds-ICCOM, 56124, Pisa, Italy; beatrice.campanella@pi.iccom.cnr.it (B.C.); onor@pi.iccom.cnr.it (M.O.); riky.46@hotmail.it (R.N.)

[2] Department of Chemistry and Industrial Chemistry, University of Pisa, 56124 Pisa, Italy; tommaso.lomonaco@unipi.it

[3] Hematology Unit, Department of Oncology, University of Pisa, 56100 Pisa, Italy; edobenedetti@gmail.com

* Correspondence: bramanti@pi.iccom.cnr.it; Tel.: +39-050-315-2293

Table S1. Sigma Aldrich product codes of standard products employed. Dilution of the stock solution were prepared in water.

compound	*Product code*	*Solubilization medium*
Creatinine	C-4255	Water
5-aminovaleric	123188	Water
Formic acid	F-0507	Water
Malic acid	240176	Water
Pyruvate	P-2256	Water
Valine	V-0500	Water
Lactic acid	07096	Water
Acetic acid	51791	Water
α-ketoglutaric acid	K-3752	Water
Dihydrouracil	D-7628	0.05 M NaOH
Uric acid	U-0881	0.05 M NaOH
Citric acid	96068	Water
GSH	G-4626	Water
Fumaric acid	47910	Water
cis-Aconitic acid	A-3412	Water
Succinic acid	S-3674	Water
Acetoacetic acid	A.8509	Water
Tyrosine	T-1145	Water
GSSG	G-6529	Water
Propionic acid	402907	Water
Phenylalanine	78019	Water
Butyric acid	19215	Water
Tryptophane	93659	Water

Table S2. Retention times, fitting parameters (slope and standard deviation SD of the slope), correlation coefficients of the calibration plots and limit of detection (LOD) of the selected metabolites analysed (V_{inj}=5 µL; N=3 replicates).

compound	*Retention time (min)**	*slope*	*SD*	R^2	*LOD (µM)*
Creatinine	3.561	9876	49	0.9999	0.02
5-Aminovaleric acid	3.636	57	2.0	0.9960	2.31
Formic acid	3.723	63	0.1	0.9999	2.07
Malic acid	4.018	162	0.3	0.9999	0.80
Pyruvate	4.272	959	1.6	0.9999	0.14
Valine	4.36	66	0.1	0.9999	1.97
Lactic acid*	4.697	110	0.3	0.9999	1.18
Acetic acid	4.935	46	0.1	0.9999	2.83
α-ketoglutaric acid	5.141	1351	2.4	0.9999	0.10
Dihydrouracil	5.167	4529	112	0.9988	0.03
Uric acid	5.358	12773	24	0.9999	0.01
Citric acid	5.496	345	1.5	0.9999	0.38
GSH	5.813	2062	14	0.9998	0.06
Fumaric acid	6.091	20940	29	0.9999	0.006

cis-Aconitic acid	6.618	16129	143	0.9998	0.008
Succinic acid	6.747	88	0.9	0.9996	1.49
Acetoacetic acid	6.978	70	7	0.9819	2.06
Tyrosine	7.476	16717	25	0.9999	0.008
GSSG	8.158	5402	158	0.9974	0.02
Propionic acid	10.059	51	0.1	0.99999	2.55
Phenylalanine	17.019	3328	5	0.9999	0.039
Butyric acid	22.890	66	0.2	0.9999	1.97
Tryptophane	23.356	63966	362	0.9998	0.002

*For lactic acid the figures of merit were comparable with those obtained previously [1]. For the other analytes, the coefficient of variation (CV%) of the measurements performed on the same and different vial was < 3%.

(1) Biagi, S.; Ghimenti, S.; Onor, M.; Bramanti, E. Simultaneous Determination of Lactate and Pyruvate in Human Sweat Using Reversed-Phase High-Performance Liquid Chromatography: A Noninvasive Approach. Biomed. Chromatogr. 2012. https://doi.org/10.1002/bmc.2713.

Table S3. Statistics on the compounds identified and quantified (mM) in saliva sample of subject n. 2 (intra-day reproducibility test, N = 3) of Figure 1.

	Min	Max	**Mean**	**SD (n-1)**	**CV% (n-1)**	Mean SE
Creatinine	0.0031	0.0034	**0.0033**	**0.0001**	**4.2**	0.0001
Formic acid	0.4444	0.4683	**0.4550**	**0.0121**	**2.7**	0.0070
Malic acid	12.191	12.747	**12.469**	**0.2778**	**2.2**	0.1604
VAL	0.1591	0.1697	**0.1626**	**0.0061**	**3.8**	0.0035
Lactic acid	0.3909	0.4045	**0.3955**	**0.0079**	**2.0**	0.0045
Acetic acid	1.5978	1.9457	**1.7935**	**0.1779**	**9.9**	0.1027
α-ketoglutaric acid	0.0089	0.0100	**0.0095**	**0.0006**	**5.9**	0.0003
Uric acid	0.1910	0.1946	**0.1925**	**0.0018**	**1.0**	0.0011
Citric acid	0.1354	0.1420	**0.1394**	**0.0036**	**2.6**	0.0021
GSH						<LOD
Fumaric acid	0.0024	0.0027	**0.0026**	**0.0001**	**5.8**	0.0001
Cis-aconitic acid	0.0007	0.0007	**0.0007**	**0.0000**	**5.3**	0.0000
Succinic acid	0.1875	0.1989	**0.1913**	**0.0066**	**3.4**	0.0038
Aceto-acetic acid						<LOD
TYR	0.0085	0.0088	**0.0087**	**0.0002**	**2.1**	0.0001
GSSG						<LOD
Propionic acid	0.1618	0.1745	**0.1699**	**0.0071**	**4.2**	0.0041
PHE	0.0074	0.0078	**0.0076**	**0.0002**	**2.6**	0.0001
Butyric acid	4.2273	4.3409	**4.2778**	**0.0579**	**1.4**	0.0334
TRP	0.0026	0.0028	**0.0027**	**0.0001**	**3.5**	0.0001

Table S4. Statistics on the compounds identified and quantified (mM) in saliva pool sample (inter-day reproducibility test, N = 3) (subject n. 4 pool).

	Min	Max	**Mean**	**SD (n-1)**	**CV% (n-1)**	Mean SE
Creatinine	0.0134	0.0138	**0.0136**	**0.0002**	**1.6**	0.0001
Formic acid	0.6825	0.7063	**0.6958**	**0.0121**	**1.7**	0.0070
Malic acid	53.648	55.275	**54.575**	**0.8368**	**1.5**	0.4831
VAL	7.7348	8.2197	**8.0227**	**0.2549**	**3.2**	0.1472
Lactic acid	4.5182	4.6727	**4.6136**	**0.0834**	**1.8**	0.0482
Acetic acid	20.044	20.6413	**20.431**	**0.3361**	**1.6**	0.1941
α-ketoglutaric acid	<LOD	<LOD	**<LOD**	**<LOD**	**<LOD**	<LOD
uric acid	0.1948	0.2223	**0.2121**	**0.0151**	**7.1**	0.0087
Citric acid	<LOD	<LOD	**<LOD**	**<LOD**	**<LOD**	<LOD
GSH	0.0318	0.0342	**0.0333**	**0.0013**	**3.8**	0.0007
Fumaric acid	0.0422	0.0435	**0.0430**	**0.0007**	**1.6**	0.0004
cis-aconitic acid	<LOD	<LOD	**<LOD**	**<LOD**	**<LOD**	<LOD
Succinic acid	0.7216	0.7443	**0.7330**	**0.0114**	**1.6**	0.0066
aceto-acetic acid	1.5357	1.5571	**1.5452**	**0.0109**	**0.7**	0.0063
TYR	0.0386	0.0398	**0.0393**	**0.0006**	**1.6**	0.0004
GSSG	0.0846	0.0880	**0.0866**	**0.0018**	**2.1**	0.0010
Propionic acid	0.7255	0.8922	**0.8301**	**0.0911**	**11.0**	0.0526
PHE	0.1107	0.1140	**0.1124**	**0.0017**	**1.5**	0.0010
Butyric acid	3.9242	4.4848	**4.1717**	**0.2860**	**6.9**	0.1651
TRP	0.0106	0.0111	**0.0109**	**0.0003**	**2.6**	0.0002

Table S5. Statistics on the compounds identified and quantified (mM) in saliva samples (N = 3) (subject n. 4).

	Min	Max	**Mean**	**SD (n-1)**	**CV% (n-1)**	Mean SE
Creatinine	0.0244	0.0319	**0.0284**	**0.0037**	**13.1**	0.0022
Formic acid	1.6429	1.8730	**1.7937**	**0.1307**	**7.3**	0.0754
Malic acid	50.389	62.732	**57.6451**	**6.4510**	**11.2**	3.7245
VAL	2.9242	5.9167	**4.4773**	**1.4994**	**33.5**	0.8657
Lactic acid	1.4273	4.4273	**2.8576**	**0.0000**	**52.7**	0.8688
Acetic acid	17.348	27.7391	**22.8986**	**5.2319**	**22.8**	3.0207
α-ketoglutaric acid	<LOD	<LOD	**<LOD**	**<LOD**	**<LOD**	<LOD
Uric acid	0.2287	0.3797	**0.3106**	**0.0763**	**24.6**	0.0441
Citric acid	<LOD	<LOD	**<LOD**	**<LOD**	**<LOD**	<LOD
GSH	0.0248	0.0436	**0.0340**	**0.0094**	**27.7**	0.0054
Fumaric acid	0.0196	0.0360	**0.0264**	**0.0086**	**32.5**	0.0050
cis-aconitic acid	<LOD	<LOD	**<LOD**	**<LOD**	**<LOD**	<LOD
Succinic acid	0.5795	1.2614	**0.9242**	**0.3410**	**36.9**	0.1969
Aceto-acetic acid	0.6786	0.8571	**0.7929**	**0.0992**	**12.5**	0.0573
TYR	0.0245	0.0426	**0.0324**	**0.0093**	**28.6**	0.0053
GSSG	0.0234	0.0359	**0.0311**	**0.0067**	**21.6**	0.0039
Propionic acid	0.5098	1.2549	**0.8072**	**0.3946**	**48.9**	0.2278
PHE	0.0616	0.1289	**0.0908**	**0.0345**	**38.0**	0.0199
Butyric acid	3.2197	6.7576	**4.5530**	**1.9231**	**42.2**	1.1103
TRP	0.0055	0.0118	**0.0086**	**0.0031**	**36.8**	0.0018

Table S6. Statistics on the compounds identified and quantified (mM) in saliva samples (N = 3) (subject n. 5).

	Min	Max	**Mean**	**SD (n-1)**	**CV% (n-1)**	Mean SE
Creatinine	0.0568	0.0850	**0.0702**	**0.0142**	**20.2**	0.0082
Formic acid	3.7143	5.9048	**4.6878**	**1.1153**	**23.8**	0.6439
Malic acid	26.228	43.253	**33.260**	**8.8902**	**26.7**	5.1327
VAL	7.2045	9.6439	**8.3030**	**1.2376**	**14.9**	0.7145
Lactic acid	3.2227	6.1773	**4.6424**	**1.4806**	**31.9**	0.8548
Acetic acid	0.0141	12.717	**7.6786**	**6.7464**	**87.9**	3.8950
α-ketoglutaric acid	0.0000	0.0000	**0.0000**	**0.0000**	**0.0**	0.0000
uric acid	0.1899	0.2761	**0.2274**	**0.0442**	**19.4**	0.0255
Citric acid	0.0000	0.0000	**0.0000**	**0.0000**	**0.0**	0.0000
GSH	0.0003	0.0003	**0.0003**	**0.0000**	**0.0**	0.0000
Fumaric acid	0.0564	0.0816	**0.0718**	**0.0135**	**18.8**	0.0078
cis-aconitic acid	0.0000	0.0000	**0.0000**	**0.0000**	**0.0**	0.0000
Succinic acid	0.0074	0.1989	**0.1275**	**0.1046**	**82.1**	0.0604
aceto-acetic acid	0.5429	1.0071	**0.8238**	**0.2471**	**30.0**	0.1426
TYR	0.0427	0.0626	**0.0519**	**0.0100**	**19.3**	0.0058
GSSG	0.0448	0.0771	**0.0562**	**0.0181**	**32.2**	0.0104
Propionic acid	4.2157	9.9412	**6.9902**	**2.8668**	**41.0**	1.6552
PHE	0.1496	0.3547	**0.2330**	**0.1078**	**46.2**	0.0622
Butyric acid	1.6061	3.3432	**2.3215**	**0.9082**	**39.1**	0.5243
TRP	0.0092	0.0131	**0.0111**	**0.0020**	**17.8**	0.0011

Table S7. µM concentration values of metabolites quantified in 13 different saliva samples form nominally healthy volunteers.

saliva sample #	*Creatinine*	*Formic acid*	*Malic acid*	*VAL*	*Lactic acid*	*Acetic acid*	*Alfa keto glutaric acid*	*Uric acid*	*Citric acid*	*GSH*	*Fumaric acid*	*Cis-aconitic acid*	*Succynic acid*	*Aceto-acetic acid*	*TYR*	*GSSG*	*Propionic ac*	*PHE*	*Butyric acid*	*TRP*
1	2.3	137.3	9321.0	-	254.6	1380	4.1	114.3	<LOD	<LOD	3.4	<LOD	51.1	<LOD	3.6	<LOD	107.8	10.2	106.1	0.6
2*	3.3	455.0	12469	162.6	395.5	1794	9.5	192.5	139.4	<LOD	2.6	0.7	191.3	<LOD	8.7	<LOD	161.8	7.6	4277.8	2.7
3	5.0	169.0	16400	1091	190.0	2270	14.4	322.0	<LOD	<LOD	1.1	<LOD	424.7	<LOD	11.5	<LOD	197.9	8.9	370.0	2.5
4*	28.4	1794	57645	4477	2858	22899	<LOD	310.6	<LOD	34.0	26.4	<LOD	924.2	792.9	32.4	31.1	807.2	90.8	4553.0	8.6
5*	70.2	4688	33260	8303	4642	7679	<LOD	227.4	<LOD	<LOD	71.8	<LOD	127.5	823.8	51.9	56.2	6990	233.0	2321.5	11.1
6*	9.7	1066	9731.5	2119	1638	2402	<LOD	95.6	<LOD	<LOD	7.4	<LOD	1199	<LOD	11.4	<LOD	1474	14.3	1371.2	2.1
7	29.9	1714	7290.1	9.8	1000	989.1	<LOD	126.2	<LOD	32.8	7.4	1.6	<LOD	<LOD	41.6	19.1	1392	42.8	13621.2	16.2
8	27.6	1968	29173	4121	8041	1130	<LOD	193.3	<LOD	44.1	1.1	1.4	<LOD	<LOD	81.0	8.3	1186	46.9	6030.3	13.1
9	10.0	277.8	32386	9.8	2368	2163	<LOD	358.9	<LOD	<LOD	13.6	<LOD	<LOD	<LOD	13.2	10.4	333.3	36.4	9.8	0.5
10	3.7	436.5	23367	9.8	2112	282.6	<LOD	222.0	<LOD	<LOD	4.7	<LOD	267.0	164.3	9.8	7.0	509.8	13.2	9.8	2.0
11	24.1	1706	356756	2705	1273	3410	<LOD	248.2	<LOD	<LOD	85.7	25.9	4756	<LOD	8.1	8.4	23.5	12.9	3047.7	5.8
12	9.7	373.0	15537	2599	354.5	14.1	<LOD	101.0	<LOD	<LOD	3.5	<LOD	233.0	<LOD	6.4	6.6	12.7	0.2	9.8	1.5
13	70.9	4278	30247		6955	9033	65.9	216.5	289.9	<LOD	7.9	<LOD	1295.5	<LOD	59.2	<LOD	44.1	25.6	2189.4	14.3

(*) mean value

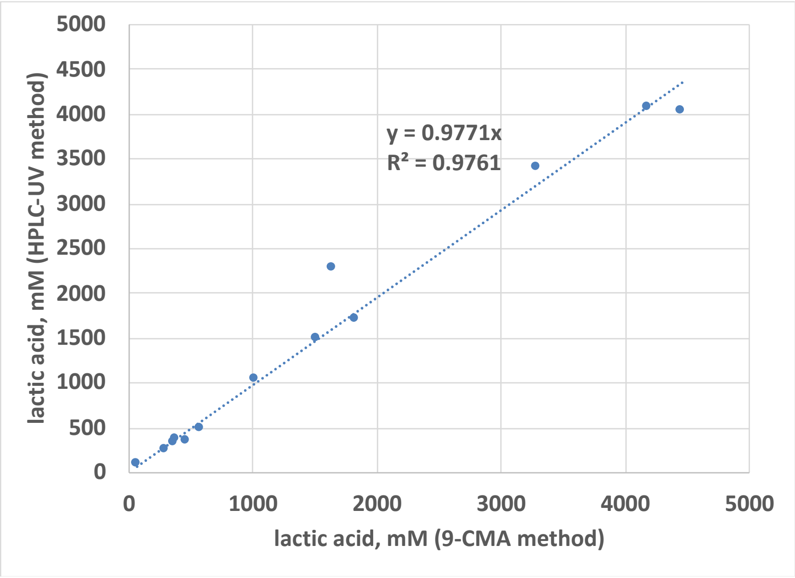

(A)

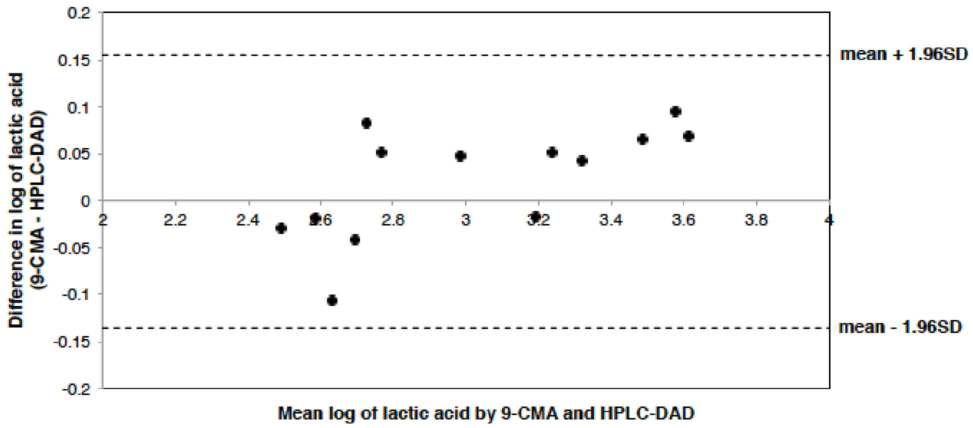

(B)

Figure S1. (A) Determination of lactic acid in saliva by 9-CMA method vs HPLC-UV method. (B) Bland-Altman plot.

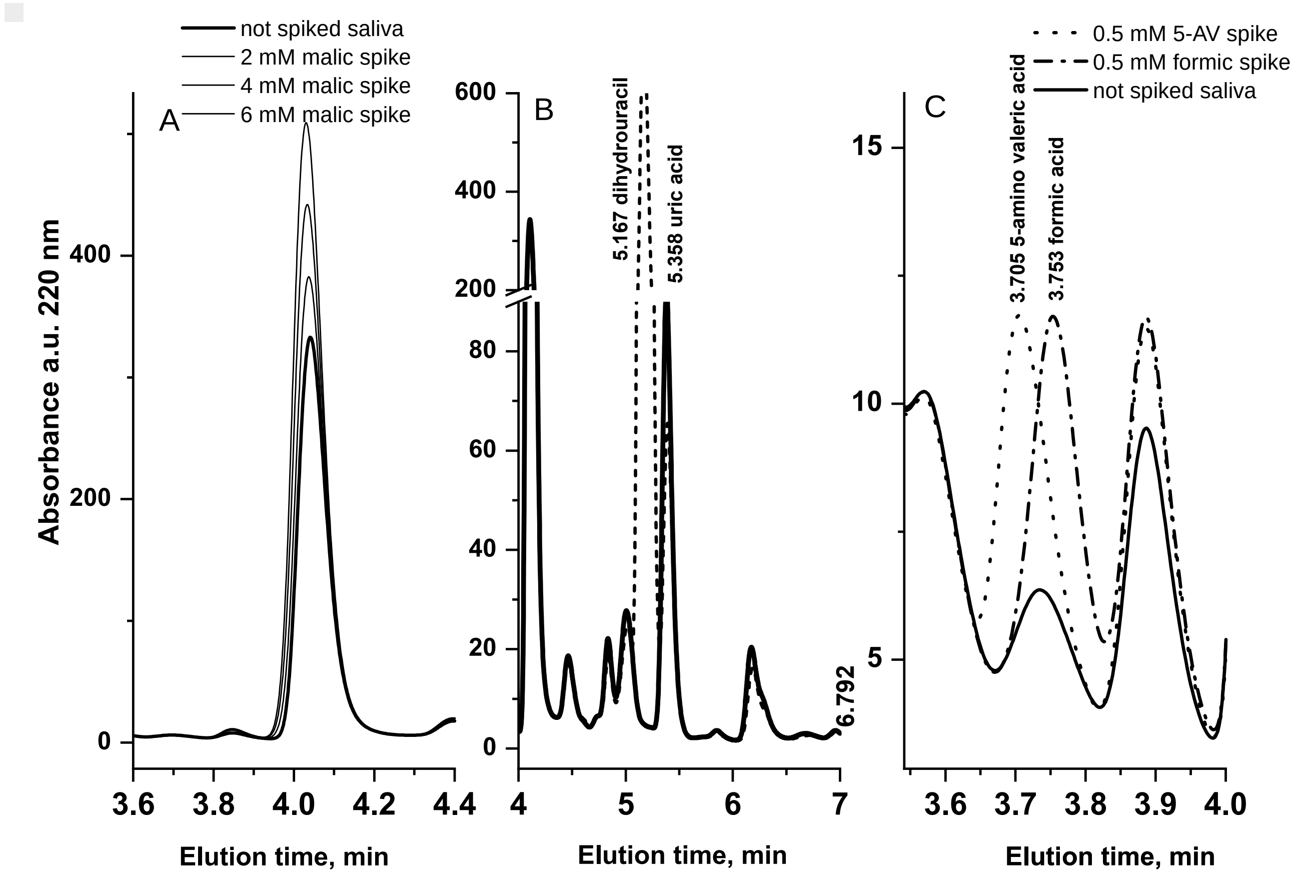

Figure S2 (A) Absorbance chromatogram at 220 nm of unspiked saliva (saliva pool n. 4) and spiked with 2, 4 and 6 mM malic acid. (B) Absorbance chromatogram at 220 nm of unspiked saliva (saliva pool n. 4) and spiked with 1 mM dihydrouracil. (C) Absorbance chromatogram at 220 nm of unspiked saliva (saliva pool n. 4) and spiked and 0.5 mM 5-amino valeric acid (dotted line) or 0.5 mM formic acid (dash-dot line)

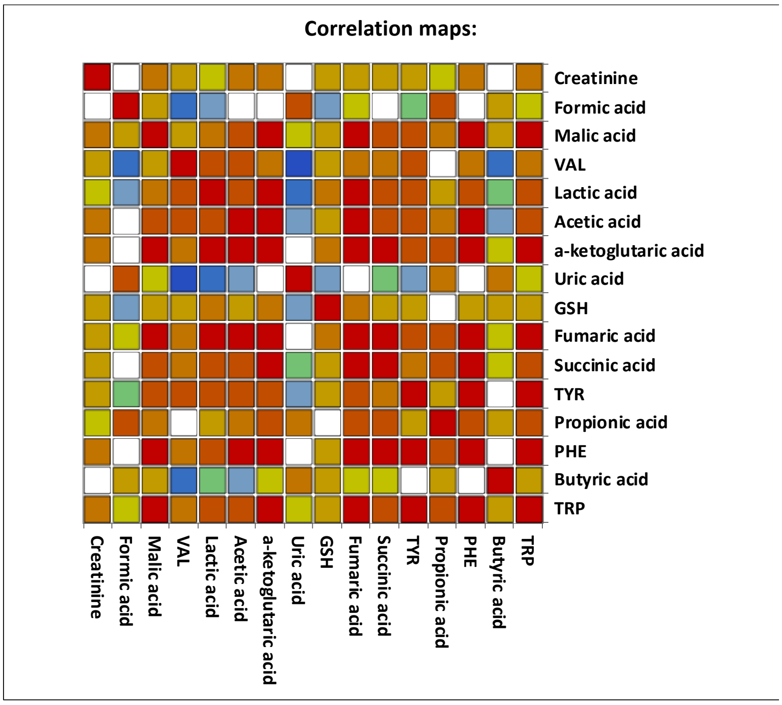

Figure S3 Correlation plot for 16 metabolites quantified in saliva samples from the experiment with rifaximin (correlation obtained after data scaling).

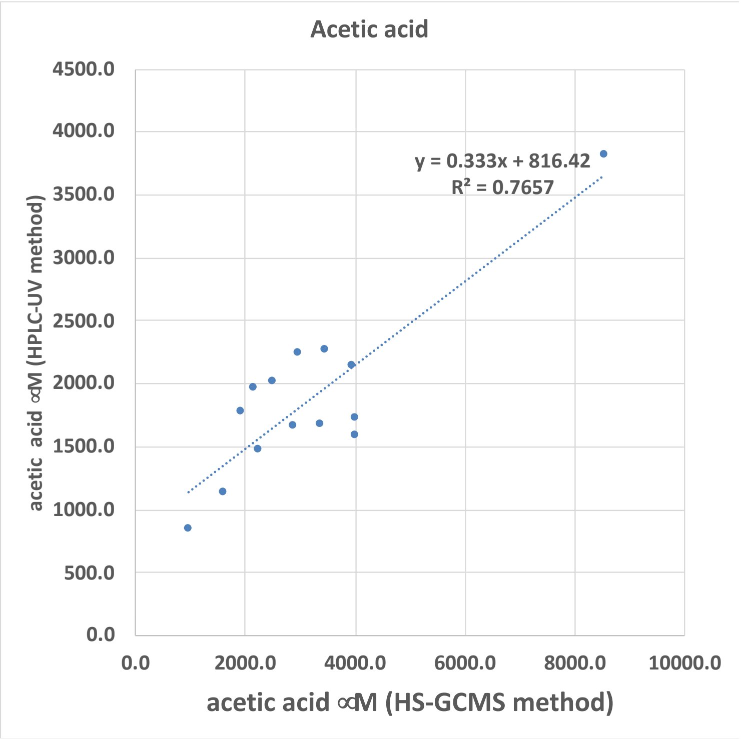

Figure S4. Determination of acetic acid in saliva by HS-GCMS method vs HPLC-UV method.

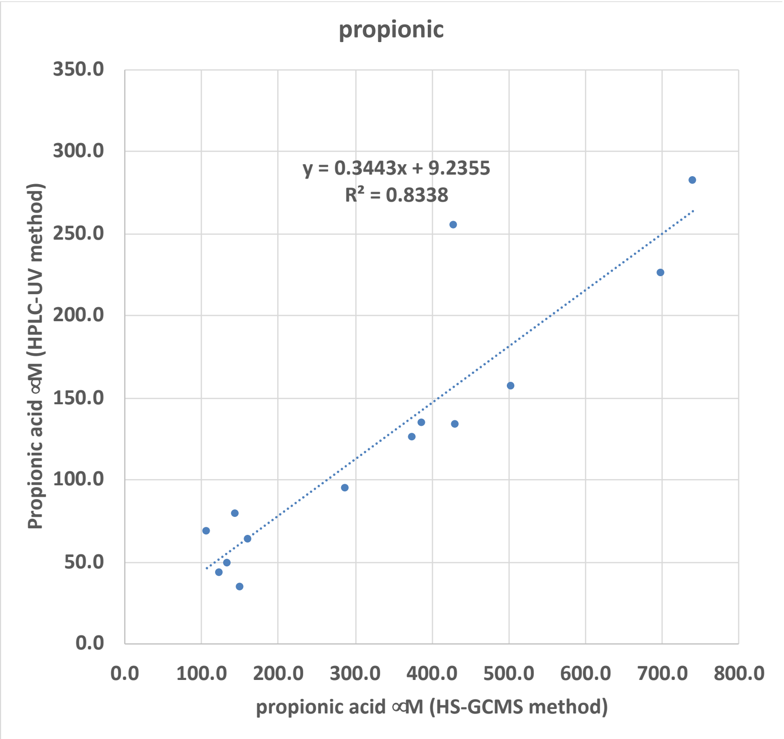

Figure S5. Determination of propionic acid in saliva by HS-GCMS method vs HPLC-UV method.
